# Factors Affecting Age at ASD Diagnosis in UK: No Evidence that Diagnosis Age has Decreased Between 2004 and 2014

**DOI:** 10.1007/s10803-016-2716-6

**Published:** 2016-03-31

**Authors:** Denise Brett, Frances Warnell, Helen McConachie, Jeremy R. Parr

**Affiliations:** Institute of Health and Society, Newcastle University, Newcastle upon Tyne, UK; Institute of Neuroscience, Newcastle University, Newcastle upon Tyne, NE1 4LP UK

**Keywords:** Autism, Autism spectrum disorder, ASD, Age at diagnosis

## Abstract

Clinical initiatives have aimed to reduce the age at ASD diagnosis in the UK. This study investigated whether the median age at diagnosis in childhood has reduced in recent years, and identified the factors associated with earlier diagnosis in the UK. Data on 2134 children with ASD came from two large family databases. Results showed that the age of ASD diagnosis has not decreased. The median age of diagnosis of all ASDs was 55 months. Factors associated with earlier age of diagnosis were autism diagnosis (compared with other ASD), language regression, language delay, lower socioeconomic status, and greater degree of support required. Effective clinical strategies are needed to identify children with characteristics that have in the past delayed ASD diagnosis.

## Introduction

There is a growing body of evidence to suggest that early intervention programmes can improve overall functioning, social communication, language, cognition and adaptive behaviour in children with autism spectrum disorder (ASD) (e.g. Magiati et al. 2014; Oono et al. 2013). As children get older, treatments may be less effective (Harris and Handleman 2000), highlighting the importance of early ASD diagnosis leading to timely intervention. Whilst ASD can reliably be diagnosed as early as 24 months (Steiner et al. 2012; Johnson et al. 2007), population based studies have found that the median age of diagnosis tends to be at around school entry age (Shattuck et al. 2009). In the UK, a number of clinical initiatives have aimed to improve ASD diagnostic services for children. For example, after widespread variation in clinical diagnostic services was found in 2001, the National Autism Plan for Children (NAP-C) was published, providing clear and structured recommendations around the identification, assessment, diagnosis and access to early intervention for preschool and primary school age children with an ASD (NAP-C 2003). Subsequently, Palmer et al. (2011) surveyed 243 UK child development teams regarding their diagnostic practices, and compared responses with 2001 data. Positive developments included an increase in the availability of multidisciplinary team (MDT) professionals, increase in the number of teams with a written ASD assessment protocol and increased use of standardised diagnostic measures. However, only one-third of teams had a defined timescale for completion of assessment, and of those, only 49 % met the recommended NAP-C timescale of completion of the process of assessment and diagnosis in fewer than 30 weeks. In 2011, the National Institute for Health Care Excellence (NICE) published guidelines on the recognition, referral and diagnosis of autism in children and young people from birth to 19 years (NICE 2011).

Despite this focus on robust and improved diagnostic assessment processes, there has been no up to date information about whether the mean or median age at ASD diagnosis of UK children has reduced. The most recent population-based children’s UK study reported a median age of diagnosis for all ASDs as 82 months (Williams et al. 2008). In a survey of 1047 parents in the UK, Crane et al. (2015) reported that the mean age of ASD diagnosis was 89 months; however 4 % of the children of these parents were over 18 years old when they got their diagnosis, with the maximum age being 40 years old. In the United States, data from 1420 children with ASD revealed that the mean age of diagnosis was 62.8 months (Oswald et al. 2015). Centers for Disease Control and Prevention (CDC) data from 2014 established that the median age of earliest known ASD diagnosis was 53 months. In a recent review of the current literature on age at diagnosis (42 UK and non-UK studies), Daniels and Mandell (2013) reported combined estimates of median age at diagnosis for all ASDs to range from 36 to 82 months.

Researchers have previously highlighted that child, family and environmental factors are associated with age at diagnosis. For example, type of ASD diagnosis is made at widely different ages. Williams et al. (2008) found the UK median age of diagnosis of ‘autistic disorder’ to be 44.9 months, 115.9 months for Asperger syndrome, and 75.5 months for Pervasive Developmental Disorder—Not Otherwise Specified/Autism Spectrum Disorder. Likewise, Daniels and Mandell (2013) found that almost half of studies reviewed reported a significantly later age of diagnosis for Asperger syndrome, compared to all other ASDs. Developmental regression has consistently been associated with earlier diagnosis (Rosenberg et al. 2011; Shattuck et al. 2009), as has having a sibling with ASD, having lower level communicative function and higher socioeconomic status (SES) (Valicenti-McDermott et al. 2012; Mandell et al. 2005; Fountain et al. 2011). However, additional neurological and psychiatric comorbidities have been shown to be associated with a later age at diagnosis (Levy et al. 2010). There is contradictory evidence for the role of other phenotypic factors in influencing age at diagnosis. Studies have not consistently shown that learning/intellectual disability and sex are linked to earlier or later diagnosis (Rosenberg et al. 2011; Shattuck et al. 2009; Frenette et al. 2013; Wiggins et al. 2006; Coo et al. 2012). Other factors such as ethnicity, maternal age, parental concern, geographical location, and proximity to specialists have all been reported as associated with age at diagnosis (Daniels and Mandell 2013). However, due to differences in study methodologies, inadequate study sizes and non-representative sampling frames, there is wide variability in findings associated with earlier or later diagnosis.

Recent multivariate analysis studies have examined which combination of child, family and environmental factors are the most strongly associated with the age at ASD diagnosis in childhood. Bickel et al. (2015) observed that significant predictors of earlier age at diagnosis were later birth order, higher maternal education, fewer children in the house, and a sibling with ASD. Furthermore, in their sample of 315 children younger than 3 years, earlier diagnosis was associated with higher cognitive and adaptive functioning, lower language level and having a sibling with ASD. Mazurek et al. (2014) found that lower age, higher SES, more severe autism symptoms and lower IQ were associated with earlier age at diagnosis; however higher functioning children were being diagnosed earlier than in previous years.

This study aimed to (1) Explore whether the median age at diagnosis in the UK has reduced in the last decade in a large and representative sample of children with ASD; and (2) Investigate the phenotypic factors associated with age at diagnosis, to identify potential groups for whom clinicians might develop additional strategies to reduce the age of diagnosis in the future.

## Methods

The data were extracted from two large ASD family research databases and included parent report of age of diagnosis for 2134 children aged 2–18 years. The Database of Children with ASD Living in the North East of England (Dasl^n^e) covers six areas around Newcastle, whilst the Autism Spectrum Database-UK (ASD-UK) covers the rest of the geographical areas of the UK. Families join one database or the other, based on their location.

### Recruitment to Dasl^n^e

Dasl^n^e’s recruitment methods since 2003, and data about the validity and representativeness of the ASD diagnoses of included children, have been described previously (McConachie et al. 2009; Wood et al. 2015; Warnell et al. 2015). In brief, families were recruited primarily through community child health and mental health teams. Parents/carers were invited to join Dasl^n^e shortly after their child received an ASD diagnosis. Following informed consent, parents completed a paper or online parent questionnaire (www.daslne.org/). The child’s diagnostic status was validated by a questionnaire completed by their clinician. Capture–recapture methods were used to ensure as many local families as possible were approached about Dasl^n^e. Validation of children’s ASD diagnoses was previously examined by selecting 40 children at random with corroboration of diagnosis using standardised assessment measures or clinical notes (McConachie et al. 2009).

### Recruitment to ASD-UK

Following ethical and local approval, recruitment of families commenced in 2011 through a network of 72 ‘research interested’ UK neurodisability, community child health and mental health teams. Families with one or more children, aged 2–16 years, who had been given a clinical ASD diagnosis, were eligible for recruitment. ASD-UK participation was discussed at a clinic appointment, or clinicians wrote to families no longer reviewed in clinic. Families received an information sheet and expression of interest form; those who responded were telephoned by ASD-UK staff to explain the project. A pack was then sent that included a consent form, parent questionnaire and the Social Communication Questionnaire—lifetime version (Rutter et al. 2003). Alternatively, parents could register and complete the consent and parent questionnaire online (www.asd-uk.com). Families could also self-refer by contacting ASD-UK directly or via the website. Families that have joined ASD-UK are broadly representative of families of children with ASD in the UK, and children included on the databases have valid ASD diagnoses (Wood et al. 2015; Warnell et al. 2015).

ASD-UK and Dasl^n^e share similar methodologies and collect parallel data. Both ask parents questions about their child with an ASD including their sex, type of ASD diagnosis [autism, ASD (synonymous with Pervasive Developmental Disorder-Not Otherwise Specified), and Asperger syndrome], language, age at diagnosis (in months), presence of a sibling with ASD, and presence of an additional diagnosis (dyslexia, dyspraxia, ADHD, learning/intellectual disability or ‘other’, in which parents can describe the additional diagnosis). ASD-UK further measures some variables historically associated with age at diagnosis, including language regression (with or without skill regression—referred to as ‘language regression’ from here on) or skill regression (with or without language regression—referred to as ‘skill regression’ from here on), parent rating of level of support required, presence of a relative with an ASD, and the presence of broader autism phenotype (BAP) type traits in other family members, categorised as having relatives with similar or milder behaviours to those seen in ASD. ASD-UK also measures SES by using the Townsend Index of Deprivation (Townsend et al. 1988). This involves assigning a measure of deprivation to families based on their postcode; the measure of deprivation for each postcode is based on unemployment, non-car ownership, non-home ownership and household overcrowding in that area.

### Statistical Analysis

All data analyses were performed using SPSS 22.0. Median age at diagnosis for children born in each calendar year was calculated using descriptive statistics. To explore whether there was a significant difference within categories of factors associated with age at diagnosis, Mann–Whitney U tests (for variables with two categories) or Kruskal–Wallis tests (for variables with more than two categories) were performed. Non-parametric tests were chosen as some of the data were non-normally distributed. Hierarchical linear regression was performed to determine variables that predicted age at diagnosis. Standardised regression coefficients are reported for linear regression analyses, with beta values reporting the relative change between categories within factors in age at diagnosis. For dummy coded variables, this was the difference between each category and the reference category. All other statistical analyses were descriptive in nature, including the mean, SD, median and interquartile range and number of children in the analysis.

## Results

**Fig. 1:**
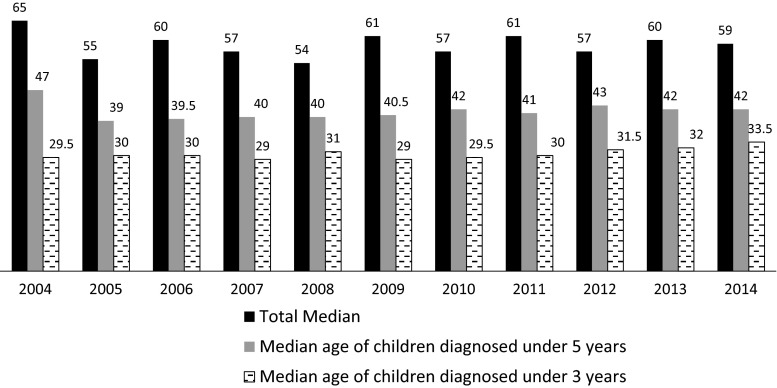
Median age at ASD diagnosis (in months) from 2004 to 2014

Data from 2134 children and families were available (1164 from ASD-UK and 970 from Dasl^n^e). 82.7 % of the included children were male, 17.3 % were female. The median age at diagnosis was 55 months for all ASDs. Age at diagnosis ranged from 7 to 223 months. Table 1 presents data on the age of diagnosis given to children in the years from 2004 to 2014. There was no significant difference in age of diagnosis across these years (*p* = .504). There was also no significant difference in the median age at diagnosis for children diagnosed under age 36 months or under 60 months (see Fig. 1). Thus, there was no evidence of a significant reduction in the UK mean or median age at ASD diagnosis over the last decade.

**Table 1:** Median age of ASD diagnosis (in months) per year, and the number and proportion of children diagnosed under 36 months and under 60 months

Year	N	Mean	SD	Median	Interquartile range	N under 36 months	% diagnosed under 36 months	N under 60 months	% diagnosed under 60 months
2004	117	72.14	30.35	65.00	39.00	6	5.1	44	37.6
2005	104	63.67	34.38	55.00	53.00	23	22.1	56	53.8
2006	101	70.76	37.24	60.00	57.00	16	15.8	42	41.6
2007	108	68.37	35.86	57.00	54.00	12	10.5	56	49.1
2008	138	70.21	39.03	54.00	59.00	22	15.2	73	50.3
2009	147	70.05	40.15	61.00	43.00	21	13.7	70	45.8
2010	215	70.45	38.74	57.00	53.00	38	17.1	115	51.8
2011	290	72.24	39.35	61.00	56.00	38	12.8	140	47.1
2012	278	74.10	42.82	57.00	54.00	36	12.7	146	51.2
2013	247	74.53	41.15	60.00	54.00	28	11.1	121	48.0
2014	47	76.48	40.82	59.00	69.00	6	12.8	23	48.9

### Associations Between Age at Diagnosis, and Child and Family Characteristics

Information on the number of children, their sex, diagnosis, language ability, learning/intellectual disability, other additional diagnoses, regression, support needed, and presence of relatives with ASD and BAP are presented in Table 2, and statistically significant results noted. The variables for which there was no significant difference in mean age of diagnosis were sex (*p* = .315), epilepsy (*p* = .861), sibling with ASD (*p* = .976) and family member with ASD (*p* = .307).

**Table 2:** Phenotypic factors and their relation to age at diagnosis (in months). Factors are grouped according to whether they were included in models 1 or 2 for the regression analyses

	N	Percent	Age of diagnosis			Interquartile range	Difference
			Mean	SD	Median		
*Model 1 factors*							
Sex							*p* = .315^a^
Male	1765	82.7	67.27	37.11	55.00	47.00	
Female	369	17.3	72.05	42.97	55.00	60.00	
Diagnosis							*p* < .001^b^
Combined	2134	100.00	68.10	38.22	55.00	48.00	
Autism	459	21.61	47.98	28.06	40.00	22.00	
ASD	1288	60.64	67.81	37.31	54.00	45.00	
Asperger’s syndrome	377	17.75	93.38	36.82	87.00	56.00	
Language							*p* < .001^b^
Verbal	1173	55.46	81.73	38.42	74.00	56.00	
Echoing/single words	632	29.88	53.99	30.41	45.50	22.00	
Non verbal	310	14.66	45.12	28.97	36.00	18.00	
Learning/intellectual disability							
Yes	737	34.54	61.14	37.23	48.00	37.00	*p* < .001^a^
No	1397	65.46	71.77	38.24	60.00	54.00	
Other additional diagnoses							*p* = .001^a^
Yes	308	14.43	75.20	42.78	60.00	56.00	
No	1826	85.57	66.90	37.27	54.00	48.00	
*Model 2 factors*							
Language regression							*p* < .001^a^
Yes	323	28.84	50.27	29.34	41.00	18.00	
No	797	71.16	72.71	37.76	60.00	52.00	
Skill regression							*p* < .001^a^
Yes	217	19.41	58.23	32.80	48.00	33.00	
No	901	80.59	67.97	37.68	55.00	49.00	
Level of support							*p* < .001^b^
Support	404	35.63	75.49	38.75	62.50	53.00	
Substantial support	491	43.30	65.69	36.41	53.00	43.00	
Very substantial support	239	21.07	52.98	31.73	43.00	25.00	
Broader autism phenotype in relatives							*p* < .001^a^
Yes	646	57.32	70.05	38.60	58.00	52.00	
No	481	42.68	61.40	34.91	48.00	40.00	
*Additional factors*							
Sibling with ASD							
Yes	241	11.29	67.69	36.96	56.00	51.00	*p* = .976^a^
No	1893	88.71	68.15	38.38	55.00	48.00	
Other family member with ASD							
Yes	308	26.95	64.93	37.05	53.00	46.00	*p* = .307^a^
No	835	73.05	66.93	37.31	54.00	45.00	
ADHD							*p* < .001^a^
Yes	287	13.45	78.17	36.45	72.00	47.00	
No	1847	86.55	66.53	38.26	53.00	46.00	
Dyslexia							*p* < .001^a^
Yes	56	2.62	98.02	42.29	104.00	68.00	
No	2078	97.37	67.29	37.79	54.00	46.00	
Dyspraxia							
Yes	199	9.33	91.55	42.45	88.00	66.00	*p* < .001^a^
No	1935	90.67	65.68	36.93	53.00	45.00	
Epilepsy							
Yes	30	1.41	74.10	49.33	52.00	56.00	*p* = .861^a^
No	2104	98.59	68.01	38.04	55.00	48.00	

^a^Mann Whitney U test, ^b^ Kruskal–Wallis test

Children with additional diagnoses were diagnosed with ASD later than children without other diagnoses (*p* = .001). Specifically, children with ADHD (*p* < .001), dyslexia (*p* < .001) and dyspraxia (*p* < .001) were diagnosed much later than children who did not have these conditions.

The effect of SES is presented in Table 3. The Townsend deprivation score (higher score denotes more deprivation) was compared for children diagnosed before 36 months and those diagnosed from age 36 months onward; a comparison was also made for children born before 60 months, and those diagnosed from age 60 months onward. There was no significant difference in the Townsend scores of children diagnosed before 36 months, and those diagnosed after this age; however, when the threshold for diagnosis was increased to 60 months, children diagnosed before 60 months tended to have a lower SES [a higher deprivation score (M = 1.22)] than children diagnosed after 60 months (M = .32).

**Table 3:** Socioeconomic status (Townsend index)

	N	Percent	Mean	SD	Median	Interquartile range	Difference
36 month cut off							
Diagnosed before 36 months	120	15.58	.94	4.61	−.83	6.32	*p* = .641^a^
Diagnosed aged 36 months or over	650	84.42	.80	4.17	−.67	4.99	
60 month cut off							*p* = .012^a^
Diagnosed before 60 months	431	55.97	1.22	4.49	−.386	5.70	
Diagnosed aged 60 months or over	339	44.03	.32	3.86	−.089	4.08	

^a^Mann Whitney U test

NB Townsend index positive values indicate more deprivation than the UK national average

### Regression Analyses

To further explore the predictive utility of the factors associated with age of diagnosis, two hierarchical linear regression analyses were carried out with age at diagnosis as the dependent variable, and predictors chosen based on significant difference in medians of subcategories, and previous research (Table 2). Visual inspection of a histogram of the residuals of the linear regression revealed a normal distribution for both regression analyses.

In the first regression analysis, phenotypic characteristics of children from both databases were included (2107 children). Sex was entered in Step 1. The dummy coded ASD diagnoses variables (Autism, Asperger syndrome) were entered at Step 2 (children with an ‘ASD’ diagnosis were the reference category). Language delay was entered in Step 3, with verbal children being the reference category. This resulted in two dummy coded variables, echoing/single words and non-verbal. Learning/intellectual disability and other additional diagnoses were entered in Step 4.

The first block, with sex as a predictor was significant, *F*(1, 2105) = 4.67, *p* = .031, R_adj_^2^ = .002 (Table 4). Although a weak predictor, boys tended to have an earlier age of diagnosis than girls (β = −.047). The second step of the model was also significant, *F*(3, 2103) = 116.22, *p* < .001, R_adj_^2^ = .141. The addition of type of ASD diagnosis accounted for a further 14.1 % of the variance in age at diagnosis. Those with an autism diagnosis were diagnosed earlier than those with an ASD diagnosis (β = −.216), while those diagnosed with Asperger syndrome were diagnosed later (β = .258). Language delay was included in Step 3, which explained a further 8 % of the variance in age at diagnosis, *F*(5, 2101) = 119.63, *p* < .001, R_adj_^2^ = .222. Children whose language repertoire included only single words or echoing were diagnosed earlier than verbal children (β = −.247). Similarly non-verbal children were diagnosed earlier than verbal children (β = −.261). The final step of the model, including learning/intellectual disability and additional diagnoses was also significant, *F*(7, 2099) = 88.52, *p* < .001 R_adj_^2^ = .225. Whilst the inclusion of learning/intellectual disability did not significantly add to the model (β = −.012), the presence of another additional diagnosis did (β = .079); those who had additional diagnoses received their diagnosis of ASD later than those without. The total model explained 22.5 % of the variance in age at diagnosis.

**Table 4:** Results of the first regression analysis (N = 2107)

	R_adj_^2^	B	SE	Beta	t	*p*
Step 1	.002					
Sex		−4.752	2.200	−.047	−2.160	.031
Step 2	.141					
Sex		−5.970	2.043	−.059	−2.922	.004
ASD (reference) versus autism		−19.995	1.931	−.216	−10.356	.000
ASD (reference) versus Asperger		25.700	2.077	.258	12.371	.000
Step 3	.222					
Sex		−5.789	1.947	−.057	−2.973	.003
ASD versus autism		−13.314	1.899	−.144	−7.012	.000
ASD versus Asperger		17.476	2.068	.175	8.450	.000
Verbal (reference) versus echoic		−20.560	1.771	−.247	−11.606	.000
Verbal (reference) versus non verbal		−28.205	2.282	−.261	−12.360	.000
Step 4	.225					
Sex		−5.426	1.945	−.054	−2.790	.005
ASD versus autism		−13.313	1.898	−.144	−7.016	.000
ASD versus Asperger		17.083	2.084	.171	8.198	.000
Verbal versus echoic		−20.577	1.779	−.247	−11.568	.000
Verbal versus non verbal		−28.249	2.285	−.261	−12.360	.000
Learning/intellectual disability		−.979	1.607	−.012	−.609	.542
Other additional diagnosis		8.574	2.080	.079	4.121	.000

Variance explained: 22.5 %

The second regression analysis included data from ASD-UK families only; the variables entered included all the variables from regression 1 as well as the additional variables measured by ASD-UK only. These included language regression, skill regression, level of support needed, and BAP in relatives, for which complete data were available from 1041 participants. As in regression 1, sex was entered in the first step, ASD diagnosis in Step 2, language level in Step 3, and learning/intellectual disability and additional diagnoses in Step 4. Parent reported language regression and skill regression were entered in Step 5. Level of support needed was entered in Step 6 as dummy coded variables, with ‘requires support’ as the reference category. This resulted in two dummy coded variables (‘requires substantial support’ and ‘requires very substantial support’). Data about relatives with BAP were added in Step 7 (Table 5).

**Table 5:** Results of the second regression analysis (N = 1041)

	R_adj_^2^	B	SE	Beta	t	*p*
Step 1	.001					
Sex		−3.786	2.889	−.041	−1.311	.190
Step 2	.145					
Sex		−4.207	2.673	−.045	−1.574	.116
ASD (reference) versus autism		−13.440	2.727	−.145	−4.929	.000
ASD (reference) versus Asperger		32.357	2.937	.324	11.017	.000
Step 3	.225					
Sex		−4.382	2.546	−.047	−1.721	.085
ASD versus autism		−7.748	2.655	−.083	−2.919	.004
ASD versus Asperger		24.397	2.917	.244	8.365	.000
Verbal (reference) versus echoic		−18.701	2.351	−.239	−7.956	.000
Verbal (reference) versus non verbal		−28.951	3.216	−.266	−9.003	.000
Step 4	.228					
Sex		−3.947	2.545	−.042	−1.551	.121
ASD versus autism		−8.424	2.663	−.091	−3.164	.002
ASD versus Asperger		25.303	2.950	.253	8.577	.000
Verbal versus echoic		−19.354	2.368	−.247	−8.172	.000
Verbal versus non verbal		−29.353	3.215	−.270	−9.130	.000
Learning/intellectual disability		4.033	2.221	.052	1.816	.070
Other additional diagnoses		4.590	2.590	.048	1.772	.077
Step 5	.241					
Sex		−4.025	2.524	−.043	−1.595	.111
ASD versus autism		−7.718	2.648	−.083	−2.915	.004
ASD versus Asperger		24.143	2.937	.241	8.220	.000
Verbal versus echoic		−17.512	2.385	−.223	−7.342	.000
Verbal versus non verbal		−26.241	3.267	−.241	−8.033	.000
Learning/intellectual disability		4.800	2.209	.062	2.173	.030
Other additional diagnoses		4.850	2.571	.051	1.886	.060
Language regression		−10.256	2.452	−.126	−4.183	.000
Skill regression		−.395	2.670	−.004	−.148	.882
Step 6	.243					
Sex		−4.239	2.524	−.045	−1.679	.093
ASD versus autism		−6.666	2.694	−.072	−2.474	.014
ASD versus Asperger		23.956	2.942	.240	8.142	.000
Verbal versus echoic		−16.942	2.413	−.216	−7.022	.000
Verbal versus non verbal		−24.565	3.363	−.226	−7.303	.000
Learning/intellectual disability		5.515	2.237	.071	2.466	.014
Other additional diagnoses		5.416	2.591	.057	2.090	.037
Language regression		−10.158	2.451	−.125	−4.144	.000
Skill regression		.321	2.691	.003	.119	.905
Support (reference) versus substantial		−1.671	2.354	−.023	−.710	.478
Support (reference) versus very substantial		−6.299	3.093	−.070	−2.037	.042
Step 7	.243					
Sex		−4.276	2.525	−.046	−1.693	.091
ASD versus autism		−6.704	2.695	−.072	−2.487	.013
ASD versus Asperger		24.115	2.951	.241	8.171	.000
Verbal versus echoic		−17.177	2.436	−.219	−7.051	.000
Verbal versus non verbal		−24.846	3.388	−.228	−7.334	.000
Learning/intellectual disability		5.410	2.242	.070	2.413	.016
Other additional diagnoses		5.599	2.604	.059	2.150	.032
Language regression		−10.205	2.453	−.125	−4.160	.000
Skill regression		.488	2.702	.005	.181	.857
Support versus substantial		−1.698	2.355	−.023	−.721	.471
Support versus very substantial		−6.478	3.104	−.072	−2.087	.037
Relatives similar difficulties		−1.488	2.108	−.020	−.706	.480

Total variance explained: 24.3 %

The first block, with sex as a predictor was not significant, *F*(1, 1039) = 1.718, *p* = .190, R_adj_^2^ = .001. The second step of the model was significant, *F*(3, 1037) = 59.83, *p* < .001, R_adj_^2^ = .145. The addition of type of ASD diagnosis accounted for a further 14.4 % of the variance in age at diagnosis. Those with an autism diagnosis were diagnosed earlier than those with an ASD diagnosis (β = −.145), while those diagnosed with Asperger syndrome were diagnosed later (β = .324). Language delay was included in Step 3, which explained a further 8 % of the variance in age at diagnosis, *F*(5, 1035) = 161.37, *p* < .001, R_adj_^2^ = .225. Children whose language repertoire included only single words or echoing were diagnosed earlier than verbal children (β = −.239). Similarly non-verbal children were diagnosed earlier than verbal children (β = −.266). The next step of the model, including learning/intellectual disability and additional diagnoses was also significant, *F*(7, 1033) = 44.989, *p* < .001 R_adj_^2^ = .228; although their addition explained a further .3 % of the variance, neither learning/intellectual disability nor additional diagnoses significantly added to the model. Parent reported language regression and skill regression were entered in the next step, which was significant *F(*9, 1031) = 37.78, *p* < .001 R_adj_^2^ = .241, explaining an additional 1.3 % of the variance. Children with language regression were significantly more likely to have an earlier age of diagnosis than those with no language regression (β = −.126); there was no difference in the age at diagnosis between children who had skill regression and those who did not (β = −.004). The next step including the level of support required by children was also significant, *F*(11, 1029) = 31.39, *p* < .001 R_adj_^2^ = .243. Children whose parents reported needing ‘very substantial support’ received an earlier diagnosis (β = −.070). Although the next step, including relatives with BAP, was significant, *F* (12, 1028) = 28.78, *p* < .001 R_adj_^2^ = .243, it did not significantly add to the model. The whole model accounted for 24.3 % of the variance in age of ASD diagnosis.

## Discussion

This study of over 2000 children shows that the median age of ASD diagnosis in the UK has not reduced in the last decade. The study also showed no evidence of reduction in the age at diagnosis of children who received their diagnosis below age 60 months, or age 36 months. The median age at ASD diagnosis in the whole sample was 55 months, in line with figures reported by the CDC in the US (2014), but much lower than the 82 months reported by the UK study of 86 children (Williams et al. 2008), and the 66–71 months reported in a previous UK study of 267 children (Latif and Williams 2007).

In the context of increasing evidence that early intervention is likely to improve some outcomes for children and families, this finding has importance for parents and clinicians, and may have significant health economic implications, considering the costs associated with ASD in the UK (Buescher et al. 2014). We do not have information about why the age at ASD diagnosis has not reduced in the last decade; however it is likely determined by a combination of a number of systemic factors. As reported by Palmer et al. (2011), in 2007 two-thirds of UK child health ASD assessment teams did not have a defined timescale for ASD assessment. Of the one-third that did, almost half were not meeting targets set out by NAP-C (2003). It is possible that early detection of ASD symptoms is occurring but that the complex pathway to getting a diagnosis results in long delays between initial parental concern, referral, assessment and diagnosis. There may be parental delay in seeking a diagnosis; some parents may notice atypical development early in their child’s life but may wait to see if more developmental progress is made with time. Suboptimal awareness of the features of ASD in family practitioners (health visitors and General Practitioners) may also be a contributing factor, and lead to parental reassurance where referral would be more appropriate. This has been recognised as a problem by organisations leading family care in the UK (The Royal College of General Practitioners); ASD has recently been adopted as a clinical priority for General Practitioners (GPs) from 2014 until 2017 with the aim of improving the training GPs receive in the recognition of ASD. Some of these factors might explain the recent evidence from 1047 UK parents that the mean time interval from initial expression of parental concern to a health professional and ASD diagnosis was 3.6 years (Crane et al., 2015). Increased publicity and awareness about ASD, and a recognition of the broader autism spectrum, means some children are being referred for assessment at school age; indeed our sample included children who received a diagnosis up to 18 years. Whilst the referral of older children could have skewed the mean age at diagnosis, it would have been unlikely to significantly alter the median age and it would not explain the lack of reduction in age at diagnosis for the children diagnosed at under age 36 months or under 60 months.

Considering the factors associated with age at ASD diagnosis, male-sex, autism diagnosis, language regression, language delay, higher levels of required support and lower SES were associated with a younger age at diagnosis. The presence of additional diagnoses was associated with a later age at ASD diagnosis in our sample of 2107 families, but when additional variables were included in the second regression model with a smaller sample, additional diagnoses were not found to be a significant predictor. Learning/intellectual disability did not affect age at diagnosis, consistent with previous research (Fountain et al. 2011; Frenette et al. 2013). This was the first study to include having relatives with BAP as a possible predictor of age at ASD diagnosis. It could be hypothesised that because other family member display these ASD-like behaviours, the manifestation of these in their own child would trigger parental concern; however this variable did not significantly predict age at diagnosis. Contrary to our hypothesis, and previous research (for example, Coo et al. 2012), having a sibling with ASD did not result in an earlier age at diagnosis. There are several possible explanations for this. When parents have one child with ASD, they may or may not recognise ASD developmental signs in the second child, as there are frequently differences in the developmental presentation. For example, the first child may have autism and language regression, whereas the sibling’s language may have developed in line with age expectations but social communication difficulties only become clearer at school age. Parental concern expressed to professionals is sometime dismissed by explanations of the second child copying the behaviours of the first, or parents being overly concerned due to having one child already on the spectrum. Previous research has reported developmental regression as being a marker for earlier diagnosis (Shattuck et al. 2009). In this study parents reported whether their child had regressed in language, and if they lost other skills. Only language regression was predictive of age at diagnosis. The fact that skill regression was not associated with an earlier age at diagnosis may also be surprising. Previous research has shown that language regression is rarely the only type of regression; Parr et al. (2011) reported 11.4 % of children who experienced regression had non-language regression. It is likely there was variability in what parents viewed as ‘skill’ regression, and therefore it is difficult to draw conclusions from this finding.

Considering the number of studies linking higher SES to an earlier age at diagnosis (for e.g. Goin-Kochel et al. 2006), it may seem surprising that in this study children diagnosed before 60 months had lower SES than children diagnosed after 60 months. However, there is universal free healthcare access in the UK, which may lessen the impact of SES on access to services in comparison with the US and some other countries.

Our results should be seen in the context of what we know about ‘red flags’ for autism (Wetherby et al. 2004), that led to the US ALARM guidance for practitioners (Parr and Woodbury-Smith 2015). In our study, children who displayed language regression were diagnosed on average at 3 years 5 months. The fact that regression most commonly occurs in the second year of life (Parr et al. 2011) means that some children were not diagnosed with ASD for almost 2 years after they lost previously acquired language skills. Children who had only single word speech at diagnosis were diagnosed on average at 3 years 9 months. One might expect children with language regression or language delay to receive much earlier diagnoses than found in this study. Children with additional diagnoses were not diagnosed until 5 years, whilst children with ADHD were not diagnosed until 6 years. Similarly, children presenting with dyslexia or dyspraxia were diagnosed much later than children who did not have these conditions. Diagnostic overshadowing and the lack of recognition of ASD in the presence of psychiatric and neurological disorders may result in delayed intervention that might potentially ameliorate their disability and improve their outcomes (Joshi et al. 2014).

Strengths of this study include the large sample size and age range. Both databases are representative of the ASD population in the UK, and ASD diagnostic validity has been shown (Warnell et al. 2015); ASD diagnoses were confirmed by medical reports supplied by parents. We also analysed a large range of variables, including child, family and environmental characteristics, using multiple regression models whereas previous studies have analysed predictors using odds ratios (e.g. Valicenti-McDermott et al. 2012). Limitations of this study include the exclusion of some variables that have previously been linked to age at diagnosis: child’s age at first parental concern, maternal age, maternal education, previous relationship with services; data on these aspects were not collected. Secondly, our data are parent-reported and the detail not corroborated by clinician report, and therefore possibly subject to recall bias; however, this should be seen in the context of retrospective parent data being a valuable and reliable source of information by clinicians evaluating child development in health clinics.

### Implications of this Research

The age at ASD diagnosis has not decreased in the UK in recent years despite increased publicity, clinical initiatives and awareness of ASD, and the knowledge that some phenotypes are strongly associated with ASD. For children, delayed diagnosis can result in lack of early intervention, suboptimal school placement, and lack of access to the strategies helpful for children with ASD. For parents, delays in diagnosis mean they are missing out on understanding their child’s difficulties, and receiving the appropriate support, help and management strategies they need (NICE 2013; Myers and Johnson 2007). So what can be done to reduce the age at ASD diagnosis? Children with phenotypic characteristics that are ‘red flags’ for ASD such as language regression and language delay could be identified through primary and other health service based intervention initiatives to accelerate the ASD diagnostic process. For children diagnosed at age 5 years or later, girls receive their diagnosis at a later age than boys, and timely diagnosis should be a focus for improvement in clinical teams. Clear understanding of the reasons for the lag in diagnosis in girls is a clear future research priority (Petrou et al., manuscript in preparation). For children who do not display these ‘red flags’, it is vital that we develop innovative clinical research strategies to ensure children and parents can access diagnostic assessment in a timely fashion.
